# Implementing eScreening technology in four VA clinics: a mixed-method study

**DOI:** 10.1186/s12913-019-4436-z

**Published:** 2019-08-28

**Authors:** James O. E. Pittman, Niloofar Afari, Elizabeth Floto, Erin Almklov, Susan Conner, Borsika Rabin, Laurie Lindamer

**Affiliations:** 1 VA Center of Excellence for Stress and Mental Health, 3350 La Jolla Village Dr., San Diego, CA 92161 USA; 2 Department of Psychiatry, University of California San Diego, 9500 Gilman Dr, La Jolla, CA 92093 USA; 3 VA Roseburg Health Care System, 913 NW Garden Valley Blvd, Roseburg, OR 97470 USA; 4 Gallup Inc., 901 F Street, NW, Washington, DC 20004 USA; 5 Department of Family Medicine and Public Health, University of California San Diego, 9500 Gilman Dr, La Jolla, CA 92093 USA

**Keywords:** Technology, Health information technology, eScreening, Implementation, Veterans, Mixed methods

## Abstract

**Background:**

Technology-based self-assessment (TB-SA) benefits patients and providers and has shown feasibility, ease of use, efficiency, and cost savings. A promising TB-SA, the VA eScreening program, has shown promise for the efficient and effective collection of mental and physical health information. To assist adoption of eScreening by healthcare providers, we assessed technology-related as well as individual- and system-level factors that might influence the implementation of eScreening in four diverse VA clinics.

**Methods:**

This was a mixed-method, pre-post, quasi-experimental study originally designed as a quality improvement project. The clinics were selected to represent a range of environments that could potentially benefit from TB-SA and that made use of the variety eScreening functions. Because of limited resources, the implementation strategy consisted of staff education, training, and technical support as needed. Data was collected using pre- and post-implementation interviews or focus groups of leadership and clinical staff, eScreening usage data, and post-implementation surveys. Data was gathered on: 1) usability of eScreening; 2) knowledge about and acceptability and 3) facilitators and barriers to the successful implementation of eScreening.

**Results:**

Overall, staff feedback about eScreening was positive. Knowledge about eScreening ranged widely between the clinics. Nearly all staff felt eScreening would fit well into their clinical setting at pre-implementation; however some felt it was a poor fit with emergent cases and older adults at post-implementation. Lack of adequate personnel support and perceived leadership support were barriers to implementation. Adequate training and technical assistance were cited as important facilitators. One clinic fully implemented eScreening, two partially implemented, and one clinic did not implement eScreening as part of normal practice after 6 months as measured by usage data and self-report. Organizational engagement survey scores were higher among clinics with full or partial implementation and low in the clinic that did not implement.

**Conclusions:**

Despite some added work load for some staff and perceived lack of leadership support, eScreening was at least partially implemented in three clinics. The technology itself posed no barriers in any of the settings. An implementation strategy that accounts for increased work burden and includes accountability may help in future eScreening implementation efforts.

Note. This abstract was previously published (e.g., Annals of Behavioral Medicine 53: S1–S842, 2019).

**Electronic supplementary material:**

The online version of this article (10.1186/s12913-019-4436-z) contains supplementary material, which is available to authorized users.

## Background

The use of health information technology (HIT) to support the provision of health care is rapidly increasing [1, 2]; yet, the evidence regarding its effect on patient outcomes is mixed [3]. There is, however, a growing body of literature supporting the use of technology to automate patient self-report health screening [4–8].

Computerized self-assessments have been successfully introduced in a variety of populations, including older adults with and without cognitive impairment [9–11], pregnant women [12], and youth [13]. Feasibility of using technology for the collection of patient-reported data has been demonstrated in many medical and psychiatric disorders [8, 13–17]. The utility of technology-based assessment is robust across numerous settings, such as hospitals, community clinics, outpatient clinics, home, and in clinical trials [6, 18, 19]. Computer-based self-assessment has been shown to have benefits for patients, providers, and systems and has shown feasibility, ease of use, efficiency, and cost savings [11, 20–26]. Thus, tablet-based self-assessments, which have the added benefits of portability and accessibility, have the potential to increase access to and the quality of healthcare.

Serving over 9 million veterans each year [27], the Veterans Administration (VA) has developed several technology-based solutions to improve the delivery of healthcare to its growing population [28–33]. The VA Center of Excellence for Stress and Mental Health (CESAMH) and VA Center for Innovation (VACI) developed the eScreening program. The eScreening program is a technology-based, self-screening tool that has shown promise for the efficient and effective collection of mental and physical health information in healthcare clinics that collect self-report data to triage care [23]. It is a web-based program designed to collect Veteran self-report information and standardized screens, such as the posttraumatic stress disorder checklist (PCL-5); alert clinicians to safety concerns; read and write to the electronic medical record; and provide veterans with personalized feedback. It is designed to tailor assessments to the specific needs of the clinic. A pilot study that compared eScreening to paper screening with post-9/11 veterans in the transition care management program found that eScreening improved accessibility, rate of screening completion, and some clinical processes, and both veterans and providers indicated satisfaction with the tablet-based assessment [23].

Despite the significant need and ample support for technology-based solutions to aid health care delivery, implementation of HIT has been challenging [34–37]. Challenges include the complex nature of technology-based interventions and the health care delivery context as well as limited understanding of what mechanisms and contextual factors influence adoption and implementation. To assist in the future implementation and scale-up of eScreening, we conducted a mixed method, quality improvement project (QIP) of the implementation of eScreening in a diverse set of clinical environments in four VA clinics using the Consolidated Framework for Implementation Research (CFIR) as a guide to identify relevant implementation constructs. We assessed leadership and clinical staff regarding technology-related, as well as individual- and system-level factors, that might influence the adoption and implementation of the eScreening intervention. The purpose was to gather information on: (1) implementation of eScreening; (2) knowledge about and acceptability, perceived fit/adaptability, and relative advantage of using eScreening; (3) usability of eScreening; and (4) facilitators and barriers to successful implementation of eScreening including system and organizational readiness and resources.

## Method

This was a mixed-method, pre-post quasi-experimental study related to eScreening implementation in four clinics at the VA San Diego Healthcare System (VASDHS) that was conducted as part of a QIP. The main objective of the QIP was to implement eScreening in four diverse clinic settings with input from frontline clinical staff and leadership.

### Contracted agency

VASDHS contracted Gallup Inc., an organization that provides research and analytics to help measure, monitor, and improve outcomes for government and non-government partners. Gallup has expertise in linking employee opinions and beliefs to the successful implementation of new processes and procedures. Gallup staff (co-author SC) was involved in the development of the interview guides, conducted all interviews and focus groups, collected quantitative survey data, completed analyses, and provided written data summaries.

### Participating clinics

The four VASDHS clinics were selected to represent a range of environments that could potentially benefit from technology-assisted data collection and from the variety of eScreening functions. Clinics widely differed on many factors, including type of services provided, goal for eScreening use, size and organization, and work flow and volume. The clinics, described in Table 1, included: 1) Transition Care Management (TCM), 2) Primary Care (PC), 3) Posttraumatic Stress Disorder (PTSD), and 4) Mental Health Access (MHA). TCM provides comprehensive screening for Veteran’s enrolling for healthcare. Primary Care provides ongoing general healthcare, PTSD provides evidence based psychotherapy treatment for trauma-related disorders, and MHA provides same day urgent walk-in clinic for mental health patients.

**Table 1:** Participating Clinics

Clinic	Type	Goal	Appointment	Patient Type	Provider type	When eScreening occurred	Person overseeing eScreening
TCM	VHA Enrollment	Demo, medical and admin data; Triage	Walk-in	Post-9/11 Veterans	Social work providers	Waiting for appointment	Social work
PC	Medical Care	Medical data; Clinical reminders	Scheduled	All era Veterans	Physicians, nurses	Prior to appointment	Admin staff
PTSD	Specialty MH Care	Symptom severity	Scheduled	All era Veterans/w PTSD	Psychologists	Prior to and during appointment	Admin staff and providers
MHA	Urgent MH Care	Symptom severity; Triage	Walk-in	All era Veterans in MH crisis	Psychiatrist and social work provider	**	**

Note: *Admin* Administrative, *Demo* Demographic, *MH* Mental Health, *MHA* Mental Health Access, *PC* Primary Care, *PTSD* Post Traumatic Stress Disorder, *TCM* Transition Care Management, *VHA* Veterans Health Administration, ** No eScreening completed

### Implementation strategy

This study began as a QIP with limited resources to support the implementation process. Therefore, the **implementation strategy** consisted of one eScreening education meeting and one hands-on training session followed by ongoing technical assistance upon request. During the education session, our implementation team provided a general overview of eScreening and the processes involved, discussed the potential benefits to staff and patients, and answered staff questions. During the second meeting, we provided hands-on training with the eScreening program using test patients. During the technical assistance sessions, we addressed more specific individual problems and concerns. All staff involved in using eScreening in each clinic were trained. In consultation with staff leadership, the types and number of assessments were selected to meet the particular needs of the clinic. In person, telephone, and email technical assistance was provided when requested throughout the implementation period by the eScreening support staff, but no data on the nature and amount of contact the eScreening team had with each clinic was collected due to resource limitations. The duration of the implementation period was 17 months, from September 2014 through January 2016.

### Data collection

**Leadership and non-leadership** staff from each VASDHS clinic were invited to participate in **interviews or focus groups**, respectively, led by Gallup staff. Individual interviews were conducted with leadership to accommodate schedules. Focus groups were conducted separately for all staff in each clinic at a time intended to maximize participation of all clinic positions (e.g., health providers, nursing, and administrative staff). Gallup conducted fourteen 30-min interviews with leadership and three 60-min pre-implementation focus groups with non-leadership personnel and across TCM, PC, PTSD, and MHA clinics in July 2014. They conducted twelve 45-min post-implementation interviews with leadership and four 90-min post-implementation focus groups with ten non-leadership personnel in February 2016. All interviews and focus groups were audio recorded.

**Implementation outcomes** were measured by usage and survey data. The number of eScreening assessments completed by each program during the 6 months after implementation was extracted from the eScreening system. Self-report level of implementation was collected via the eScreeening survey.

In addition to the focus groups, Gallup collected **survey data from non-leadership staff** post-implementation. After post-implementation focus groups were conducted non-leadership staff was given a unique identifier and access code to take an anonymous online web survey. Staff had 2 weeks to complete the online survey.

### Instruments

The **interview guides** (see Additional files 1, 2, 3 and 4) **for the individual and focus group interviews** were developed using the Consolidated Framework for Implementation Research (CFIR) [38]. CFIR is a broadly used, comprehensive implementation science model that includes constructs associated with successful implementation in the literature. In our interview guide we focused on the inner and outer setting, with questions such as, “Tell me how leadership has communicated that changes made today to implement eScreening will affect the organization in the future?” and “How will veterans adapt to the new process?”. We also asked questions related to the characteristics of intervention and individual domains of CFIR such as, “What is the first thing that comes to your mind when you hear the term eScreening?”; “What are your expectations for eScreening?”; and “I use eScreening in the clinic”. The **online survey** was a combination of the **Gallup Employee Engagement Survey** (Q12®) and an **eScreening-specific survey**. The Q12® is regularly used by Gallup to measure employee engagement and has been validated to predict workplace performance [39]. The Q12® contains 12 items that measure employees’ basic needs (expectations and materials and equipment), individual-level factors (accomplishment, recognition, importance and development), team-level items (contribution, mission, connection, and work ethic) and opportunity for growth and progress [39]. Each of the 12 items are scored on a Likert scale (1–5, strongly disagree to strongly agree), analyzed separately, and then combined for “Engagement” and “Satisfaction” scores. The means are compared to a relevant normative group (Government Workgroup-Level database) to determine percentiles for comparison.

An investigator-created, eScreening-specific 9-item survey assessed confidence in leadership, staff ability to adapt to changes, opinions about training, concerns about technical issues, perception of barriers, satisfaction with the implementation process, and eScreening use. Each item was scored individually on a Likert scale (1–5, strongly disagree to strongly agree). Item examples include: “Technical issues or problems with eScreening were resolved quickly” and “I am satisfied with the eScreening implementation process in the clinic”.

### Data analysis

Pre- and post- implementation focus groups and individual interviews were recorded and transcribed verbatim. A Gallup staff member, who is also a co-author (SC), analyzed the data to identify common concepts and themes that emerged from the CFIR-informed interview guide. Domains were selected a priori and focused on inner and outer setting and characteristics of intervention and individuals. Reports of summarized findings and recommendations by CFIR domain and clinic group (post-implementation only) were prepared. These data were further analyzed by the investigators (LL, JP, BR) and categorized once consensus was achieved according to the project aims: knowledge about and acceptability, perceived fit/adaptability, and relative advantage of using eScreening; usability of eScreening; and facilitators and barriers to successful implementation of eScreening, including system and organizational readiness and resources.

Descriptive analyses of the Q12® data collected were performed using Gallup’s proprietary software to yield mean scores for each clinic for the 12 individual survey items and the “Engagement” and “Satisfaction” subscales. The means were compared to a relevant normative group (Government Workgroup-Level database) to determine percentiles for comparison. Higher percentile scores are associated with stronger performance, such as increased productivity. Descriptive analyses of the eScreening specific questionnaire were calculated to compare means for each item by clinic.

In order to **compare results of qualitative and quantitative data in combination**, the pre-post qualitative results were categorized as either positive, mostly positive, mixed, mostly negative, or negative. The quantitative and qualitative data were cross tabulated to assess for congruence or difference.

## Results

### Participants

To ensure anonymity in this relatively small sample, demographic characteristics of leadership staff beyond their organizational role were not collected. In the pre-implementation interviews and focus groups of non-leadership participants, there were 10 licensed independent providers, 9 nurses, and 13 medical support staff. There were 9 pre-implementation interviews with leadership. Post-implementation interviews and focus groups consisted of all non-leadership personnel and included 13 licensed independent providers, 5 nurses, and 16 medical support staff.

### Implementation of eScreening

The number of eScreening assessments collected over the 6 months post-implementation was 1026 for TCM, 337 for PTSD, and 113 for PC clinics. The MHA clinic conducted no screening assessments. On the study-specific survey, clinics rated their use of eScreening on a scale of 1–5, with higher scores indicating greater use. The TCM clinic reported the highest level of use (M = 4.5, SD = .58), followed by PTSD (M = 3.8, SD = .84), and PC (M = 3.6, SD = 1.15). Clinics self-report of degree of implementation of eScreening were consistent with the number of tablet-based assessments completed. These two data sources were combined to categorize implementation level: the TCM was considered fully implemented, PTSD and PC were grouped as partially implemented, and MHA had no implementation.

### Interviews and focus groups

#### Knowledge about and acceptability of using eScreening

##### Perceived fit

Nearly all participants used optimistic language in **anticipation of the new electronic tool** that would replace paper screening.

*It will be helpful in terms of streamlining the process for veterans – and for staff to do the more mundane administrative tasks that we have to do.* (Pre-Implementation).

Most participants perceived the goals for eScreening as **patient-centered**. They believed it would be distinctly **superior to collecting veterans’ health information on paper** because of the ability to quickly identify problem areas and initiate treatment for veterans. eScreening was seen as particularly **valuable in emergency situations** requiring swift interventions, such as suicidal patients.

Participants also believed that eScreening would **aid the integrity of the information collected from veterans**. A few participants said that veterans would receive **more personalized care** because eScreening will capture baseline information upon entry into the VA system, important demographics, and offer the medical providers big-picture data that can be trended over time.

*I think it can help identify sets of problems that might not otherwise be detected through face-to-face interviews – but where the patient might get beneficial treatment. I think eventually it can lead to more personalized care where we could individualize their treatment plan a bit more based on the pattern of responses*. (Pre-Implementation).

Post-implementation perspectives on the perceived fit of eScreening varied according to the amount of implementation. The MHA clinic staff, which never implemented eScreening, were concerned about the **severity of psychiatric symptoms** exhibited by veterans attending their clinic **interfering with data collection**, the possible **negative effect of eScreening on the clinical encounter**, and the feasibility to use eScreening in the context of the rapidly growing volume of veterans served. Participants believed that an eScreening program would be a poor fit for a MHA clinic because it serves as triage for “gravely disabled, suicidal, and homicidal” veterans that is more appropriately done through a face-to-face appointment.

*It’s not advisable for our emergency clinic because our numbers are going up for various reasons and to implement a new tool, it’s not the right time. We used to see at most 25 veterans in our clinic per day; now we can see upwards of 40. To implement a new tool without someone in charge of it, I don’t think that’s feasible. If it populates, I need to see [the patient] right away and it makes me responsible; what if this guy said on [the iPad] that ‘I am suicidal?’ it’s important to have that face-to-face conversation*. (Post-Implementation, MHA Clinic Provider).

Another disadvantage described by participants was the **interference of data entry** by the veteran during the clinical encounter. Staff in primary care also noted that the **inconsistent use of eScreening** among veterans did not result in a clinic-wide benefit of the technology.

*We don’t use it enough to say that [it helps track the patient’s care any better]. I don’t think above and beyond what the information that would have been garnered if my LVN or myself had done the clinical reminder*. (Post-Implementation, PC Clinic Provider).

##### Relative advantages and impact

Prior to implementation, many staff participants predicted eScreening’s **usefulness in tracking trends** that could improve the VA healthcare system over the long term.

*To expedite accessing care. If patients are putting in mental health concerns and they’re screening positive, then those get in quickly, consults are quickly referred,* etc. *I think it’s an impetus for looking at patterns and trends to see some of the problem areas.* (Pre-Implementation).

*It’s quicker. It expedites a patient’s ability to get assessed by somebody – to get linked with the services that they need*. (Pre-Implementation).

Following implementation, eScreening was considered to have a **positive impact in the delivery of clinical care** In the TCM clinic. The other clinics viewed the impact of **eScreening as mixed**. **Triaging care and the efficiency of data capture** were the most prominent positive aspects of eScreening reported. Real time information, ease of use, and increased completion rates were also mentioned as benefits of eScreening.

eScreening’s ability to triage care in the TMC clinic was described as a significant advantage. Participants noted that the rapid turnaround of information meant that veterans who need immediate care could get the services they needed. Staff stated:

*I think it gives us real time information for veteran care on suicidality or homicidality, and so we can act on that. If we’re not available, the folks in enrollment member services are excellent at helping someone get to the services they need.* (Post-Implementation, TCM Clinic Provider).

##### Privacy

One important concern staff had prior to the implementation of eScreening involved **privacy** for veterans to complete eScreening. Concerns regarding the storage of the tablet devices were also raised.

*I worry about privacy. If they do it in the waiting room it can sometimes get fairly crowded. If they are sitting next to another patient, wedged in between other patients, I don’t know if they are going to want to answer all of these potentially sensitive questions if somebody is looking over their shoulder*. (Pre-Implementation).

*Where are we going to keep them? That’s the biggest challenge right there in my clinic*. *Where am I going to charge them?* (Pre-Implementation).

Concern about privacy were also noted post-implementation. Some staff reported that some patients were resistant to eScreening because they perceived it as impersonal or had concerns about the privacy of their electronic information.

*…, we have a lot of younger vets who don’t want to use it because they think that the government is going to steal their information. A lot of people think it’s impersonal. A lot of our veterans actually prefer the face-to-face contact with a nurse going through the questions – and not answering them on their own. It’s not the ethos of the VA to just say to the veterans ‘Do it.’* (Post-Implementation, PC Clinic Provider).

##### Logistics/workflow

Implementation of eScreening not only required the introduction of technology into the clinics, but it also **altered the logistics of the check-in processes and work flow**. Both benefits and challenges were reported. Some clinics’ staff found it possible for veterans to **complete the screening process with little or no assistance** while waiting for an appointment, and they noted that having the information available for the provider was an efficient use of time.

*Usually if they’re in the office and I’m doing enrollment, they are completing it across from me. They may ask a couple of questions and I can usually help them with it. We give them the tablet while they’re waiting for their name to be called for registration. It works out like that. It really works as a filler because we have upwards of sometimes an hour wait for enrollment*. (Post-Implementation, TCM Clinic Provider)

It was noted in other clinics, however, achieving this efficiency would require that patients arrive early for appointments and that **some assistance would be occasionally needed**. It was clear that having the provider assist the veterans with data entry mitigated the efficiency advantage of eScreening.

*I think the main challenge has always been the logistics of how you get the tool into the hands of patients in terms of how the patients arrive, the sequencing of their visit. So my sense has been that technology is probably pretty straightforward, but the processes that support it in deploying it to patients is more challenging*. (Post-Implementation, PC Clinic Provider).

*I had hoped that there would be better uptake of it by the patients, but it did kind of point out to me that over half of my patients arrive either just on time, a few minutes late, or significantly late for their appointment. So at least over 50% were not offered e-screening, as a result*. (Post-Implementation, PC Clinic Provider).

#### Usability of eScreening

##### Functionalities

Some focus group participants expressed concern that **length of screening** could be a barrier to the implementation of eScreening and that a shorter personalized set of screens for each veteran’s needs might facilitate success. Others expressed **concerns about confidentiality**.

*The frustrations for veterans might be that it’s a burdensome process where we have them doing too many measures – that might make it unsuccessful. One thing that would make it successful is not having it be a burden – having the assessment individualized,* i.e. *if we have a reason to administer a measure for a certain patient, then we select that measure, but not having every patient do twenty measures.* (Pre-Implementation).

The **capabilities of the eScreening program** were viewed positively across most participants in those clinics with at least partial implementation. The ability to customize the assessments, visually display data, and complete clinical reminders were some of the features that were favorably viewed. Staff recognized the efficiency of patient-entered data.

*I think it’s been really helpful to me is that the eScreening is set up so that it will also have the patient do clinical reminders. And I will so often forget to do those. I actually get feedback -- it says, ‘good utilization of clinical reminders.’ So that helps me in terms of my evaluation for work and efficiency. That makes it a lot easier*. (Post-Implementation, PTSD Clinic Provider).

The **tailoring of the assessment to the needs** of the clinic and the **efficiency of data capture** was noted by several of the clinics’ staff.

*The OEF/OIF screen which is a battery of symptoms that are totally not related to what you’re necessarily having the visit for. And so to have that completed in advance actually frees up a fair amount of clinic time in that visit.* (Post-Implementation, PC Clinic Provider).

##### Personal characteristics of veterans

Concerns about the usability of eScreening with older veterans and those with certain physical impairments were brought up during both pre- and post-implementation interviews. Participants believed that those most familiar with technology and digital devices, **younger veterans**, would have **fewer problems** with eScreening and will likely embrace it. Nearly all participants expressed **concern about older veterans or patients of any age who are visually or physically challenged.**

*Some of the younger veterans seem to really like it and I think just even knowing that we’re starting to integrate technology showcases that we’re actually keeping up to date. Something about that that creates a message that our healthcare is partially on the cutting edge, as opposed to here is some printed out piece of paper.* (Post-Implementation, PTSD Clinic Provider).

*We have some patients who are quite elderly and who really don’t have the skills to be able to do it without your reading it to them for whatever reason – disabilities they’re dealing with at the time or they just don’t understand how it works and they’re punching buttons and they don’t get it. There might just be some people that we can’t really integrate in and that’s OK*. (Post-Implementation, PTSD Clinic Provider).

##### Technology-related problems

Some staff noted that **communication with the VA check-in kiosks** would need to be addressed before eScreening could be fully implemented.

*The kiosk and the eScreening do not communicate; that needs to be addressed. We have patients who don’t’ want to stand in line or they’re late for their appointment and in a rush so they just go to the kiosk and that’s how we are going to miss them for eScreening*. (Post-Implementation, PC Clinic Provider).

Although there were some technical issues related to the computer tablets, internet connectivity and communication among existing electronic medical records, post-implementation interviews noted that technical assistance was readily available and that issues were quickly resolved.

#### Facilitators and barriers to the successful implementation of eScreening (including system and organizational readiness and resources)

##### Introduction and training

Participants were largely **satisfied with the introduction and training** that was provided. They also found the **technical support adequate**.

*Everyone got individualized training. When I had any follow-up questions, [name] was very responsive, as well*. (Post-Implementation, PTSD Clinic Provider).

However, the **introduction process of eScreening varied among clinics**. In particular, some staff noted that the length of time between the training on eScreening and the launch of the program was crucial to successful implementation. In those clinics where there was a long lag between training and introduction of eScreening (PC and MHA Clinics), implementation was incomplete.

*The overall buy-in was harder because, coincidentally, the teams were changing significantly throughout the process of the rollout. Those initial meetings were a really long time ago -- like 2 years ago.* (Post-Implementation, PC Clinic Provider).

##### Openness to change

**Openness to change** was identified as a facilitator for implementation by focus group participants. The TCM clinic noted that they had prepared their team for the changes associated with the implementation of eScreening.

*I was originally resistant – thought it would be extra work because the iPad would be a challenge for some of our veterans and they would come up and ask questions. So I was a little standoffish at first. Once we got it off the ground, I was able to see the ease of the program itself and the use of the tablet. In fact, I have some thoughts about expanding some of the usage possibilities.* (Post-Implementation, TCM Clinic Provider).

The two clinics with partial implementation (PTSD and PC Clinics) noted some resistance was present in some staff.

*Obviously, I think part of the problem is, of course, change.*

The MHA clinic expressed the least openness to eScreening.

*The entire idea of turning all of mental health into a screening process is a very VA-oriented type of thinking and is, in and of itself, flawed. When they try to elaborate it with these different tools and electronics and notes and documentation, you’re just compounding what was already a flawed idea in the first place. It may work in other specialties; I’m open to that, but not in mental health*. (Post-Implementation, MHA clinic staff).

##### Resources and leadership support

When asked to anticipate what types of barriers may hinder the integration of eScreening into their clinics, some focus group participants predicted the **need to hire personnel.**

*The personnel available to administer the screens is key… I think that’s the piece that is still a little tricky. We need someone to always be there to administer the screeners when they need to be administered; otherwise we’ll go back to the old paper way which really takes up more time*. (Pre-Implementation).

*I think it’s going to be difficult. The set-up is dysfunctional. If the patient has difficulty with the e-screening, they’re going to take the iPad and get back on that line to ask the clerks which is going to slow things down even more.* (Pre-Implementation).

Most staff agreed that with **adequate resources and work flow modification**, the use of eScreening would benefit veterans.

*I see a lot of potential for it being a really excellent and official tool. So I would be supportive if it was used throughout the hospital and throughout the VA, then these things would need to fall into place – and they would. It’s going be a standard of care.* (Post-Implementation, PTSD Clinic).

*I don’t know that I would find it beneficial to continue it. There are options to be creative with it and if we can find a way where it benefits everyone’s workload, then I would be in favor of continuing it.* (Post-Implementation, PC Clinic).

Though most **leadership expressed strong support** for eSceeening in individual interviews, many staff focus group participants sensed **a lack of enthusiasm for the project** either because of little to no communication from the top.

*I haven’t heard anything from my supervisor regarding eScreening tablets so I assume he knows. Maybe he’s on board; it just wasn’t communicated to us.* (Pre-Implementation).

An apparent relationship between **degree of eScreening implementation and leadership engagement** was noted. The TCM clinic was clearly aware of leadership support for the use of eScreening. In both the PC and PTSD clinics, which had partial implementation, there was confusion about leadership engagement. Moreover, some participants felt that there was insufficient accountability for the implementation of eScreening that affected its successful adoption.

*There are no consequences for not using eScreening. I would have no idea if my LVN was offering it. I have no idea if the AMSA’s up front are doing it. I feel like I’m cut off from the process a little bit. If it happens, it’s without my input.* (Post-Implementation, PC Clinic).

##### Length of screening, confidentiality, and logistical concerns

The **physical layout of the clinics** also presented some logistical challenges. Different designated spaces for check-in and waiting areas generated difficulty with the efficient use of eScreening.

*There is definitely a space problem. We have a lot of confusion. We shouldn’t have to walk down the hall, walk back down the hall – there is not enough space for the people in the front line to take care of the patients’ needs and the eScreening. We have two computers and most days we have 15–20 people nonstop for hours as walk-ins.* (Post-Implementation, PC Clinic)

Other barriers to the use of eScreening related to the **diverse demographic and clinical characteristics of veterans served** and to **inadequate staffing** to oversee the distribution of, interaction with, and collection of the computer tablets.

*I think La Jolla primary care clinic has been particularly challenging just because of our demographics. We get the sickest, the worst behaved, the oldest patients, and the walk-ins.* (Post-Implementation, PC Clinic).

### Pre- post- implementation similarities and differences

Impressions about eScreening were mostly positive at pre- and post- implementation, although some concerns related to adequate resources, changes to workflow, technical difficulties, and privacy were expressed at both time points. The impact of eScreening on clinical care was perceived as largely positive at pre-implementation, but was mixed at post-implementation for the clinics that had partial or no implementation. Concerns about the usability of eScreening with older veterans and those with certain physical impairments were brought up during both pre- and post-implementation interviews. However, at post-implementation, it was also noted that some younger veterans expressed concerns about privacy and the impersonal nature of computerized assessment. Prior to implementation, staff perceived a lack of leadership support and communication regarding eScreening. At post-implementation, the clinics with partial or no implementation also described less leadership support and engagement.

Most participants were open to using eScreening at pre-implementation. However, during post-implementation interviews, there was more resistance to change in the clinics with partial or no implementation. Training was described as a facilitator for the implementation of eScreening at both time points. Perceived barriers at pre-implementation included concerns such as the length of the assessment, privacy, and device storage. Post-implementation barriers for some included the timing of the training (e.g., extended time between training and actual implementation of eScreening), inadequate resources, changes to workflow, certain veteran characteristics (e.g., acute patients), and a few technological challenges.

### Survey results

**Fig. 1:**
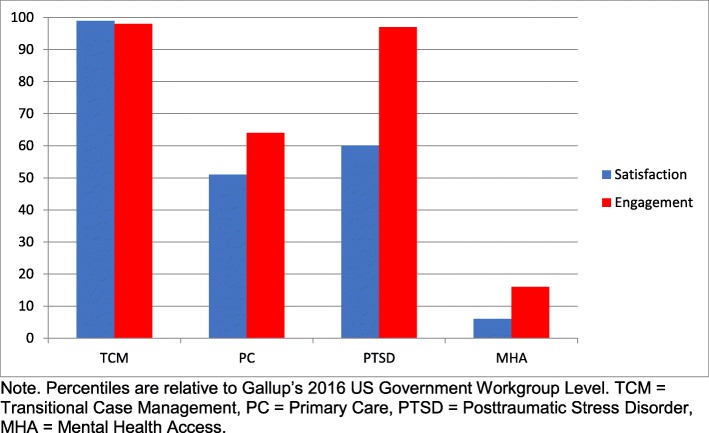
Satisfaction and Engagement scores from the Q12® by VA clinic. Graph of percentiles of Satisfaction and Engagement scores from the Q12® for each VASDHS clinic relative to Gallup’s 2016 US Government Workgroup Level

Three of the four clinics reported Engagement and Satisfaction scores above the 50th percentile relative to the Gallup’s 2016 US Government Workgroup-Level Database. The MHA clinic scored in the lower quartile for both Engagement and Satisfaction (Fig. 1).

**Fig. 2:**
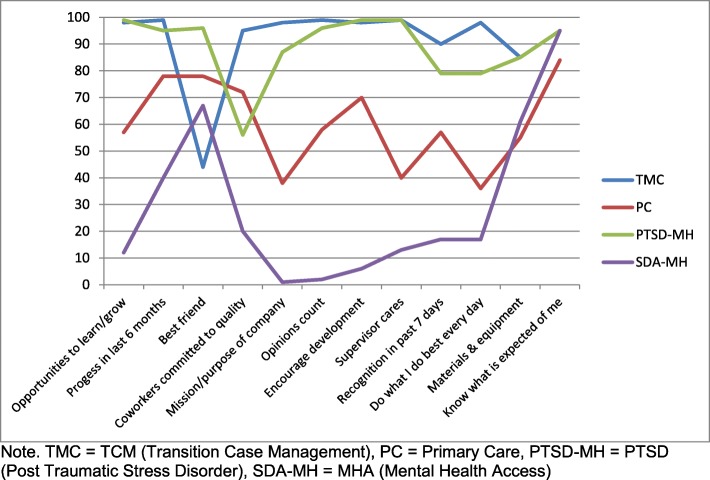
Percentile rankings of individual Q12® items by VA clinic. Graph of percentile rankings for each of the 12 individual Q12® items by VASDHS clinic

On the individual items of the Q12® Index, the TCM and PTSD clinics scored well above the 50th percentile range on nearly all the items, compared to Gallup’s 2016 US Government Workgroup-Level Database. Most of the Q12® individual items for the PC clinic were above the 50th percentile. However, the participants from the MHA clinic scored under the 25th percentile on several items related to individual and teamwork engagement factors (see Fig. 2).

Results of the post-implementation eScreening survey indicated that the TCM, PC, and PTSD clinics positively viewed the implementation of eScreening (See Table 2). There were no differences in the clinics’ views that eScreening improves healthcare, and they were equally likely to continue it as well as recommend it.

**Table 2:** Means and standard deviations from the eScreening Survey by clinic

	TCM	PC	PTSD	MHA
Confidence in facility leadership to manage challenges	3.6 (.89)	2.7 (1.19)	3.8 (.84)	2.0 (1.41)
Able to adapt when changes occur that affect my job	4.2 (.84)	4.2 (.94)	4.4 (.55)	2.8 (1.50)
Received the training necessary	4.8 (.45)	4.1 (1.80)	4.6 (.55)	*
Technical issues were resolved quickly	4.8 (.45)	3.8 (.90)	4.6 (.55)	*
Implementation improves healthcare that Veterans receive	4.6 (.56)	3.8 (1.13)	4.4 (.55)	*
Satisfied with eScreening implementation process	4.6 (.56)	3.1 (1.41)	4.4 (.55)	*
Recommends the use of eScreening for all Veterans	4.8 (.45)	3.5 (1.47)	3.8 (1.10)	*
There are significant barriers to implementing**	3.0 (1.58)	3.1 (1.39)	2.8 (1.26)	*
Use eScreening in the clinic	4.5 (.58)	3.6 (1.15)	3.8 (.84)	*

Note. *too few to score ** reversed scoring

### Integration of qualitative and quantitative findings

Clinics with higher scores on the quantitative measures showed more positive attitude toward the implementation of eScreening in the qualitative interviews. The TCM clinic, which had the highest level of eScreening implementation/usage, had the most positive attitude toward the implementation of eScreening both in the quantitative and qualitative assessments. In contrast, the MHA clinic, which did not implement eScreening, demonstrated the lowest scores and least positive attitudes toward the implementation of eScreening on both quantitative and qualitative measures. Results are summarized in Table 3.

**Table 3:** Qualitative and Quantitative Results

Data	Type	TCM	PTSD	PC	MHA
Pre	Qualitative	Positive	Mixed	Mixed	Mixed
Post	Qualitative	Positive	Mostly positive	Mixed	Negative
Survey	Quantitative				
Q12® Satisfaction		> 95%tile	> 50%tile	> 50%tile	< 10%tile
Q12®Engagement		> 95%tile	> 95%tile	> 60%tile	< 25%tile
eScreening		Positive	Mixed	Positive	Negative/missing
Usage	Quantitative	Full	Partial	Partial	None

## Discussion

The purpose of this mixed method, quasi-experimental quality improvement project of the evaluation of eScreening implementation in four VA clinics was to gather information on: (1) implementation of eScreening; (2) knowledge about and acceptability, perceived fit/adaptability, and relative advantage of using eScreening; (3) usability of eScreening; and (4) facilitators and barriers to successful implementation of eScreening, including system and organizational readiness and resources.

Results showed that implementation was variable: one clinic was successful, two were partially successful, and one was unsuccessful. Survey data on worker satisfaction and engagement, as well as readiness to change, paralleled the degree of implementation. A convergence of both quantitative and qualitative data indicated that openness to change, leadership engagement and accountability, and work flow and sufficient staff and space had a significant influence on the implementation of eScreening. The role of these internal context-related variables in facilitating or impeding implementation of evidence-based interventions has been shown broadly in the literature and more specifically in the context of the VA [40].

Knowledge on and acceptability of eScreening were generally positive. Qualitative data revealed that participants saw the clinical benefit of eScreening: the ability to triage care, capture and track clinical data efficiently and accurately, and meet reporting requirements. However, some concerns about changes to workflow were raised. In addition, at post-implementation, some concerns about fit for some populations and interference of eScreening with the clinical encounter emerged. Another concern raised by staff participants regarding the implementation of eScreening was the acuity/severity of psychiatric symptoms. The MHA clinic reported that eScreening was not a good fit for veterans with significant psychiatric symptoms. The appropriateness of computerized assessment clearly needs to be considered and weighed against the purpose of information collection for every clinic. Comprehensive screening may not be appropriate in urgent care clinics, as the MHA clinic staff commented, but brief symptom measures may be feasible. This differs from studies that have reported successful implementation of computer assisted assessments in those with severe mental illness and opioid users in the emergency department [8, 41].

Quantitative and qualitative data on usability were also generally positive. A strength of eScreening may be its flexibility in the type of information gathered. It is not an off-the-shelf system, but, rather, it is tailored to the needs of the clinic and has been developed iteratively with input from multiple stakeholder and user groups. Involvement of stakeholders in the design and development of HIT has been found to be crucial in its implementation [42]. We found that engaging providers early in the configuration of the data capture appeared to be an important component of the implementation of eScreening.

Aspects of the technology presented few challenges related to the usability and implementation of eScreening, as seen in both survey results and focus group and individual interviews. The general lack of technology-related problems may be a function of the growing familiarity of HIT both in and out of the VA, as well as the improved infrastructure and support for various technologies. Notably, while technology presented few difficulties in implementation, one exception was with older veterans and those with certain disabilities that made using eScreening difficult. Other VA studies of healthcare technology have found that both older and younger veterans easily engaged with technology, but strongly emphasize that HIT should have user-friendly features and be intuitive [29]. Although some staff participants believed that the integration of technology into healthcare delivery was inevitable and that veteran users would need to accept it, accessibility issues continue to be an important aspect of usability, and consequently, adoption. Coincidentally, VA is adopting human-centered design in the development of HIT, and researchers have found that veterans preferred standardized, integrated, and synchronized interface designs [29]. Continued input from providers and veterans regarding functions and capabilities, as well as usability of eScreening is important and appears to be instrumental in its implementation.

Facilitators of eScreening implementation included higher workplace engagement, preparation for change, and perceived leadership support. Conversely, perceived lack of leadership support and accountability, patient screening burden, physical location, and lack of personnel support were barriers. Several other factors appeared to be related with eScreening implementation. These encompassed barriers associated with the veterans, including demographic characteristics, such as age, and other medical and clinical factors like dexterity, visual acuity and severity of psychiatric symptoms. Like other studies of HIT, we found that staff reluctance to change was a barrier to the implementation [34, 36]. The MHA clinic was particularly reluctant to change. However, this may have been due, in part, to the observation that they did not seem to realize the full value and potential of the tool. The more challenging issues of the implementation of eScreening in VA clinics related to staff and physical resources and work flow, a common problem observed with the introduction of HIT [34]. eScreening clearly impacted work flow, so team approach for its implementation is important, as was demonstrated by the TCM team. Workload may increase for some members of the team, so it is important that benefits are communicated to all team members.

This study has several methodological characteristics common with quality improvement projects that challenge internal validity, such as the lack of a control condition, samples of convenience, and variation in the implementation strategy. Generalizability of these findings is limited to the sites similar to those of the current study. Clinics were not randomly selected, and, as noted from the focus group and interview data, there was no accountability for the providers to implement eScreening, thus leading to selection bias. Another limitation of the study is the inability to determine the percent and representativeness of veterans reached in each clinic because data on patient volume was not collected. The implementation strategies used in this study are well established [43], but they were not theoretically informed by a particular framework or theory and we did not collect information on the amount of technical assistance provided to each clinic. The pre-implementation quotes were organized by Gallup by CFIR construct only, so we were unable to attribute them to a specific clinic. Finally, the VA is a fully integrated healthcare system where employees have clear incentives to adhere to organizational policies in contrast to different systems where physicians are not employees. As a next step, our team will use information from the work presented in this paper and will develop a multi-component implementation strategy that will build on the improvement science methodology.

## Conclusions

This study is one of the first to use a mixed method design to assess the implementation of a computer assisted assessment system in the VA healthcare system. Although this study was a quality improvement project, the triangulation of quantitative and qualitative data strengthens credibility of results.

Perhaps the most studied example of the implementation of HIT to date is the electronic medical record (EMR), and reviews of the case studies of successes and failures of EMR implementation have highlighted the importance of context, both organizational and social factors [44]. Several frameworks have been described - socio-technology theories, contextual implementation model, triangle evaluation model - that all include multilevel factors such as “macro”, “micro”, and individual levels of influences very similar to dissemination and implementation frameworks used to guide implementation of clinical guidelines or evidence-based practices.

Despite some added work load for some staff and perceived lack of leadership support, eScreening was at least partially implemented in three of the clinics. The technology itself posed no barriers in any of the settings. An implementation strategy that that includes a strong communication and training/technical assistance plan, accounts for increased work burden and changes to work flow, and sets accountability expectations may help future eScreening implementation efforts. It may also be important to address engagement and change management for employees as part of implementation efforts, which can be accomplished through approaches such as the Lean Six Sigma Rapid Process Improvement Workshop.

## Additional files

Additional file 1: Pre-implementation Leadership Interview. (DOCX 34 kb)

Additional file 2: Pre-impllementation Focus Group Interview. (DOCX 34 kb)

Additional file 3: Post-implementation Focus Group Interview. (DOCX 37 kb)

Additional file 4: Post-implementation Leaderhip Interview. (DOCX 35 kb)

## Data Availability

The datasets supporting the conclusions of this article are available available from the corresponding author on reasonable request.

## Abbreviations

CESAMH: The VA Center of Excellence for Stress and Mental Health
CFIR: Consolidated Framework for Implementation Research
EMR: Electronic Medical Record
HIT: Health Information Technology
MHA: Mental Health Access
PC: Primary Care
PCL-5: Posttraumatic Stress Disorder Checklist
PTSD: Posttraumatic Stress Disorder
Q12: Gallup Employee Engagement Survey
QIP: Quality Improvement Project
TB-SA: Technology-Based Self-Assessment
TCM: Transition Care Management
VA: Veterans Administration
VACI: VA Center for Innovation
VASDHS: VA San Diego Healthcare System
