# Supplementary material for: Implementing eScreening technology in four VA clinics: a mixed-method study
Source: BMC Health Serv Res. 2019 Aug 28;19:604. doi: 10.1186/s12913-019-4436-z (PMC6712612; doi:10.1186/s12913-019-4436-z)
Supplement: Supplementary file 1 — Pre-implementation Leadership Interview. (DOCX 34 kb) [file 12913_2019_4436_MOESM1_ESM.docx]

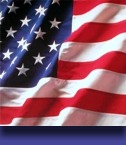
 **San Diego Veterans Administration Hospital**

**eScreening Program Pre-Implementation**

***Leadership Interview***

**San Diego VA Hospital e-Screening Program Pre-Implementation Focus Group Guide**

1. **Introductions and Ground Rules**
2. **introduction**
3. **Greeting:**

**Thank you for taking the time to speak with me today. As you know, there is a lot of attention being placed on Veterans access to and the delivery of care and services. The VA in San Diego has been a leader in forward thinking and identifying how they can improve the programs and services provided veterans at this community. We already know that the veterans value eScreening.
Know it is important step to get information from stakeholders, like yourself about what you think is important and what you think is needed to make eScreening successful. I am looking for your expertise, your thoughts, concerns and opinions regarding eScreening to help align the organization for a successful rollout. Everything you say is confidential and all information will be combined and reported in a way that prevents linking any individual to a specific comment.**

1. **Intervention Characteristics**
2. **What is the first thing that comes to your mind when you hear the term eScreening?**
3. **What is the advantage of implementing the eScreening program in your work location? For the organization overall? Explain.**
4. **How easy or difficult will it be to integrate eScreening in your work location? Explain.**
5. **What are some barriers to integrating eScreening in your work location? In the organization? Explain**

***PROBE FOR: People barriers, environmental barriers.***

1. **What do you see as solutions to the barriers you identified?**
2. **What will you do to help overcome identified barriers?**

1. **Outer Setting**
2. **How will the implementation of eScreening impact the care and services provided to veterans?**
3. **What additional policies, strategies or incentives need to be considered or implemented to support eScreening?**
4. **Inner Setting**
5. **Hospitals are known to be political organizations. What is the current political environment surrounding eScreening?**
   - **Is the change to eScreening perceived as a necessary change?**
   - **Is the current state conducive to integrating eScreening into the organization? Explain.**
   - **Will eScreening support the values of this organization? Which ones? Explain.**
6. **Tell me if you think your group is ready to implement a major change like eScreening. Explain your position.**
   - **If they are not, what needs to be done to prepare them?**
   - **How will you actively support the changes affecting your group?**
7. **From your perspective, how will individuals in your work environment, including you adapt to the new process?**
8. **How committed and/or supportive is leadership for eScreening?**
   - **How they hold people accountable for implementing the program?**
9. **How supportive of this change are you? Explain why or why not.**
10. **What information was requested from you about how eScreening should be implemented? Explain.**

1. **What will it take to make eScreening successful?**
   - **People perspective**
   - **Team perspective**
   - **Environment perspective**
2. **Characteristics of Individuals**

**Shifting gears a little,**

1. **What are your expectations for eScreening?**
2. **What additional information or training is needed to effectively implement eScreening?**
3. **What needs to happen to insure the implementation of eScreening is successful?**

***PROBE FOR: In individual’s work environment; in organization overall.***

1. **Closing Comments**
2. **Is there anything else that we have not discussed that you think would be important for me to know.**
3. **What are your suggestions on how any issues or concerns you have about eScreening be resolved?**
