# Supplementary material for: Implementing eScreening technology in four VA clinics: a mixed-method study
Source: BMC Health Serv Res. 2019 Aug 28;19:604. doi: 10.1186/s12913-019-4436-z (PMC6712612; doi:10.1186/s12913-019-4436-z)
Supplement: Supplementary file 2 — Pre-impllementation Focus Group Interview. (DOCX 34 kb) [file 12913_2019_4436_MOESM2_ESM.docx]

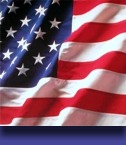
 **San Diego Veterans Administration Hospital**

**eScreening Program Pre-Implementation**

***FOCUS GROUP MEETINGS***

**San Diego VA Hospital e-Screening Program Pre-Implementation Focus Group Guide**

1. **Introductions and Ground Rules**
2. **Focus Group Moderator introduction**
3. **Greeting:**

**Thank you for taking the time to speak with me today. As you know, there is a lot of attention being placed on Veterans access to and the delivery of care and services. Veteran’s needs have been assessed and innovative ways to meet those needs have been explored. After some research and pilot testing this facility is ready to implement eScreening, a web-based software solution to aid in the process of documenting self-report health information into veteran’s medical record. Since the value of eScreening to the veteran is known as a result focus groups and pilot testing, an additional important step is getting information from stakeholders, like yourselves about what you think is important and what you need to make eScreening successful. I am looking for your expertise, your thoughts, concerns and opinions regarding eScreening in helping determine what needs to be done to align the organization for a successful rollout. Everything you say is confidential and all information will be combined and reported in a way that prevents linking any individual to a specific comment.**

1. **Ground rules:**

- **Confidentiality – what is said in the group stays in the group**
- **Respect one another**
- **Be honest and open, not looking for right or wrong**
- **Be non-judgmental**
- **Speak one at a time**
- **Inform of audio taping**

1. **Introduction of focus group participants**
   - **First Name, what you do when you come to work every day.**
2. **Intervention Characteristics**
3. **What is the first thing that comes to your mind when you hear the term eScreening?**
4. **What do you know about eScreening?**

- **What is its goal?**
- **Why is it being introduced?**
- **How does it differ from paper screening?**

1. **What information do you think would be most useful to know about eScreening at this point in time?**
2. **How easy will it be to integrate eScreening in your work location? Explain.**
3. **What are some barriers to integrating eScreening in your work location? Explain**

1. **Outer Setting**

**Let’s switch gears a little and talk for a few minutes about impact on your veterans.**

1. **How will the implementation of eScreening improve the care and services provided to veterans?**
2. **From your perspective, how will veterans adapt to the new process?**
3. **Inner Setting**

**A changing in a procedure, even when perceived as necessary and positive is still a change. I’d like to explore how changes impact this organization.**

1. **First, tell me what and how eScreening has been communicated to the organization.**
2. **Tell me how leadership has communicated that changes made today to implement eScreening brings about will affect the organization in the future?**
3. **How committed and/or supportive is leadership and management for eScreening?**
   - **Will they hold people accountable for implementing the program?**
4. **What information was requested from you about how eScreening would affect work, if at all? Explain.**

1. **Next, tell me your thoughts about the current screening procedure for new veterans - is it efficient, comprehensive and does it provide a comprehensive assessment of the new patient?**
2. **How ready is the environment in your work environment to make the change to eScreening? Explain.**
3. **How supportive of this change are you? Explain why or why not.**
4. **Next, I would like to know how new concepts have been introduced into this organization in the past?**
   - **Was the approach successful?**
   - **If not why?**
   - **What might be a better way to do it?**
5. **What will it take to make eScreening successful?**
   - **People perspective**
   - **Team perspective**
   - **Environment perspective**
6. **Characteristics of Individuals**
7. **What are organization’s the overall expectations for eScreening?**
8. **What are your expectations for eScreening?**
9. **How will eScreening be implemented in your work environment?**
10. **What additional information or training do you need to effectively implement eScreening?**
11. **What needs to happen to insure the implementation of eScreening is successful?**

***PROBE FOR: In individual’s work environment; in organization overall.***

1. **Closing Comments**
2. **Is there anything else that we have not discussed that you think would be important for me to know.**
3. **What are your suggestions on how any issues or concerns you have about eScreening be resolved?**
