# Supplementary material for: Implementing eScreening technology in four VA clinics: a mixed-method study
Source: BMC Health Serv Res. 2019 Aug 28;19:604. doi: 10.1186/s12913-019-4436-z (PMC6712612; doi:10.1186/s12913-019-4436-z)
Supplement: Supplementary file 3 — Post-implementation Focus Group Interview. (DOCX 37 kb) [file 12913_2019_4436_MOESM3_ESM.docx]

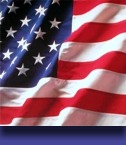
 **San Diego Veterans Administration Hospital**

**eScreening Program Post-Implementation**

***FOCUS GROUP MEETINGS***

**San Diego VA Hospital e-Screening Program Post-Implementation Focus Group Guide**

1. **Introductions and Ground Rules**
2. **Focus Group Moderator introduction**
3. **Greeting:**

**Thank you for taking the time to speak with me today. After some research and pilot testing of the eScreening tool, the facility implemented eScreening in Primary Care, Mental Health and Transition Team Clinics. The value of eScreening to the veteran was known as a result focus groups and pilot testing. The next important step is getting information from stakeholders, like yourselves, about eScreening and its use. I am looking for your expertise, your thoughts, concerns and opinions regarding the tool. There are no right or wrong answers, just insights and honest opinions. Everything you say is confidential and all information will be combined and reported in a way that prevents linking any individual to a specific comment.**

1. **Ground rules:**

- **Confidentiality – what is said in the group stays in the group**
- **Respect one another**
- **Be honest and open, not looking for right or wrong**
- **Be non-judgmental**
- **Speak one at a time**
- **Inform of audio taping**

1. **Introduction of focus group participants.**
2. **First Name, your position/job function (provider, nurse, MSA), briefly how you use eScreening in your job?**

**NOTE: Bolded questions are priority questions.**

1. **Intervention Characteristics**
2. **How did the actual eScreening tool and its use compare to your expectations?**
3. Was the eScreening tool what you thought it was?

- How did the tool meet your expectations?
- What disappointed you?

1. Did the eScreening tool meet the implementation goals?

- Increasing efficiency?
- Improving Veteran care and satisfaction with care?
- Improving the completion of clinical reminders?

1. What function of eScreening did you find most useful?
2. What function or functions were not useful?
3. How would you describe eScreening now that you have had experience with it?
4. **What facilitated your use of the eScreening tool?**
5. **What hindered your use of the eScreening tool?**
6. **Outer Setting**

**Let’s switch gears a little and talk for a few minutes about impact on your veterans.**

1. How did eScreening impact patient care?
2. From your point of view, how did the Veterans reach to eScreening?
3. What were the perceived benefits/costs, if any, did eScreening bring to your clinic?
4. **Inner Setting**

**A change in a procedure, even when perceived as necessary and positive, is still a change. I’d like to explore how eScreening has impacted your work.**

1. **First, tell me how you were approached about implementing the use of eScreening in your clinic?**
2. How was the use of eScreening introduced to your group?

- **Probe For:** Adequate training; timing; sufficient staff.

1. **How committed and/or supportive was leadership and management for eScreening?**

- **How are they holding people accountable if they don’t use eScreening?**

**PROBE FOR: *What happened if people didn’t use eScreening?***

1. **What effect did the structure of your clinic have on the implementation of eScreening?**

- **Personnel structure?**
- **Physical structure?**

1. What needs to change in your environment to increase/improve the use of eScreening?

- Additional resources?

1. Now, tell me your thoughts about the screening procedure:

- How efficient was the tool?
- How comprehensive?
- How helpful was the assessment for the clinic and the Veteran?
- How did implementation of eScreening effect your job?

1. **What problems arose during eScreening implementation? How were they handled**

- **Technical problems?**
- **People problems?**
- **Other problems?**

1. **What else needs to change in your environment to increase/improve the use of eScreening?**
2. **How supportive are you about continuing eScreening? Explain why or why not.**
3. **What might have been better or more effective way to implement eScreening in your clinic? What suggestions do you have for future implementation? Explain.**
4. **What additional resources would be needed to make eScreening successful for its current use?**

- **What is need to improve implementation in the future?**

**NOTE: *Have each participant respond.***

- - **People perspective?**
  - **Team perspective?**
  - **Environment perspective?**

1. **Characteristics of Individuals**
2. **What additional information or training do you need to effectively implement eScreening?**
3. **What needs to happen to insure the implementation of eScreening is sustained?**

**PROBE FOR: *In individual’s work environment; consistent use of tool in clinics and in organization overall.***

1. **Closing Comments**
2. **Is there anything else that we have not discussed that you think would be important for me to know.**
3. **What are your suggestions on how any issues or concerns you have about eScreening be resolved?**
