# Supplementary material for: Implementing eScreening technology in four VA clinics: a mixed-method study
Source: BMC Health Serv Res. 2019 Aug 28;19:604. doi: 10.1186/s12913-019-4436-z (PMC6712612; doi:10.1186/s12913-019-4436-z)
Supplement: Supplementary file 4 — Post-implementation Leaderhip Interview. (DOCX 35 kb) [file 12913_2019_4436_MOESM4_ESM.docx]

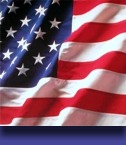
 **San Diego Veterans Administration Hospital**

**eScreening Program Post-Implementation**

***Leadership Interview***

**San Diego VA Hospital e-Screening Program Post-Implementation Focus Group Guide**

1. **Introductions and Ground Rules**
2. **introduction**
3. **Greeting:**

**Thank you for taking the time to speak with me today. After some research and pilot testing of the eScreening tool, the facility implemented eScreening in Primary Care, Mental Health and Transition Team Clinics. The value of eScreening to the veteran was known as a result focus groups and pilot testing. The next important step is getting information from stakeholders, like yourselves, about eScreening and its use. I am looking for your expertise, your thoughts, concerns and opinions regarding the tool. There are no right or wrong answers, just insights and honest opinions. Everything you say is confidential and all information will be combined and reported in a way that prevents linking any individual to a specific comment.**

1. **Intervention Characteristics**
2. **What was you experience in the implementation of eScreening in the clinics?**
3. **How did the actual eScreening tool and its use compare to your expectations?**
4. **From what you know personally, and/or what you may have heard from others, describe the eScreening implementation process?**

- **What facilitated the use of the eScreening tool?**
- **What hindered the use of the eScreening tool?**

1. Did the eScreening tool meet the implementation goals?

- Increasing efficiency?
- Improving Veteran care and satisfaction with care?
- Improving the completion of clinical reminders?

1. Outer Setting
2. How did eScreening impact patient care?
3. From your point of view, how did the Veterans reach to eScreening?
4. What were the perceived benefits/costs, if any, did eScreening bring to your clinic?
5. Inner Setting

**A change in a procedure, even when perceived as necessary and positive, is still a change. I’d like to explore how eScreening has impacted getting work done.**

1. How was the use of eScreening introduced to your group?

**Probe For:** Adequate training; timing; sufficient staff?

1. **How supportive of this change were you initially?**

- **How has you opinion changed post implementation?**
- **How supportive are you about continuing eScreening? Explain why or why not.**

1. What effect did the structure of the clinics/your clinic have on the implementation of eScreening?

- Personnel structure?
- Physical structure?

1. How well did individuals in your work environment, adapt to a new process and work flow?
2. Now, tell me your thoughts about the screening procedure:

- How efficient was the tool?
- How comprehensive?
- How helpful was the assessment for the clinic and the Veteran?
- How did implementation of eScreening effect your job?

1. **What problems arose during eScreening implementation? How were they handled**

- **Technical problems?**
- **People problems?**
- **Other problems?**

1. **What processes or steps would you change in eScreening implementation in the future?**

1. Characteristics of Individuals

Shifting gears a little,

1. What additional information or training is needed to effectively implement eScreening?
2. What needs to happen to insure the implementation of eScreening is sustained?

*PROBE FOR: In individual’s work environment; in organization overall.*

1. Closing Comments
2. Is there anything else that we have not discussed that is important for me to know.
3. What are your suggestions on how any issues or concerns you have about eScreening be resolved?
